# The Role of Pornography Use in Intimate Partner Violence in Different-Sex Couples: A Prospective Longitudinal Study

**DOI:** 10.1177/08862605211055145

**Published:** 2021-11-16

**Authors:** Katherine Jongsma, Patti Timmons Fritz

**Affiliations:** 1 7989 University Health Network, Toronto, Ontario, Canada; 2 University of Windsor, Windsor, Ontario, Canada

**Keywords:** pornography, intimate partner aggression, couple dyads, actor–partner, longitudinal

## Abstract

Pornography use and intimate partner violence (IPV) are both prevalent in romantic relationships. However, information is lacking about whether pornography use predicts IPV. This study examined the relation between frequency of pornography use (FPU) and IPV across a span of 4 months in a sample of 132 different-sex couple dyads. At least one partner in each couple was attending a Canadian university. Participants (*N* = 264) completed online measures of pornography use, IPV, and social desirability at baseline and at a 4-month follow-up. Two longitudinal actor–partner interdependence models using a structural equation framework to conduct path analyses demonstrated that (a) higher FPU among men at baseline predicted increases in IPV perpetration and victimization from baseline to 4-month follow-up for both men and women and (b) women’s baseline FPU did not predict change in IPV over time for themselves or their partners. These findings suggest that frequent pornography use among male partners in different-sex romantic relationships may represent an under-recognized risk factor for IPV, and further research is needed to identify latent factors that may be contributing to this relation. Although women’s baseline FPU did not predict changes in IPV over time, this may be because women used pornography less frequently than men.

## Introduction

Pornography use has increased as it has become more accessible on the Internet, and online pornography use is now prevalent globally. Within romantic relationships, researchers estimate that 71–80% of men and 28–59% of women use pornography (Bridges & Morokoff, 2010; Hatch et al., 2020; Minarcik et al., 2016; Poulsen et al., 2013), with men viewing pornography more often than women (Petersen & Hyde, 2010).

Intimate partner violence (IPV) is harmful physical, sexual, or emotional behaviors committed by a current or former spouse or romantic partner (Breiding et al., 2015; Dutton & Goodman, 2005), and it is also quite prevalent. A recent review of national population-based IPV estimates for women in North and South America reported that annual rates of physical and/or sexual IPV ranged from 1.1% in Canada to 27.1% in Bolivia (Bott et al., 2021). According to a national victimization survey in Canada (Lysova et al., 2019), 2.9% of men and 1.7% of women reported experiencing physical and/or sexual IPV in their current romantic relationship within the last 5 years. Among 10,565 U.S. college students, researchers reported the following proportions of men and women, respectively, who endorsed experiencing different types of IPV at least once during their time in college: 33.8% and 38.1% endorsed physical IPV perpetration, 41.9% and 32.4% endorsed physical IPV victimization, 23.0% and 13.6% endorsed sexual IPV perpetration, 27.0% and 30.1% endorsed sexual IPV victimization, 86.5% and 86.4% endorsed psychological IPV perpetration, and 86.5% and 83.0% endorsed psychological IPV victimization (Fass et al., 2008).

Assessing the relation between pornography use and intimate partner violence (IPV) is thus important given that both pornography use and IPV are prevalent in romantic relationships (Minarcik et al., 2016) and that IPV is related to such negative outcomes as poor physical and mental health among IPV perpetrators and victims (Coker et al., 2002; Fanslow et al., 2021; Lawrence et al., 2012; Lagdon et al., 2014; Stark et al., 2020) as well as children exposed to IPV (Fritz & Roy, 2020). The purpose of the current study was to examine whether pornography use contributes to IPV while accounting for romantic partners’ mutual influence on each other and their baseline levels of pornography use.

### Literature Review

The primary theory that describes the relationship between pornography use and violence is Malamuth et al.’s (1995) confluence model of sexual aggression, which provides an empirically driven theoretical account of how pornography contributes to sexual aggression in men by way of direct and indirect effects through impersonal sex and hostile sexist attitudes. It theorizes that within associative cognitive networks, more distal cognitive schemas hierarchically activate and prime more proximate cognitive constructs, which in turn, increase sexually aggressive behaviors. Baer et al. (2015) found that sex drive accounted for effects previously attributed to pornography use and that men with high hostile masculinity and sexual promiscuity were more likely to view violent pornography, which suggested that the contribution of pornography use to men’s sexual aggression needed to be re-evaluated. However, more recent work by Malamuth (Malamuth et al., 2021) found that extreme pornography use predicted sexual violence through hostile masculinity, but pornography use did not impact the relationship between impersonal sex and sexual aggression in a sample of 1148 male U.S. college and university students. The lack of support for impersonal sex as a mediator between pornography use and sexual aggression can be further understood when considering a recent study by Wright et al., (2021) which included 1016 men in a US national probability sample. As expected, pornography exposure was positively associated with sexual aggression for men who were high in impersonal sex; however, the relationship between pornography use and sexual aggression was stronger among men who were lower rather than higher in impersonal sex. When considering results from meta-analyses, the link between pornography use and sexual aggression in men and women have been mixed, with one meta-analysis finding that pornography use is associated with sexual aggression (*r* = 0.28; Wright et al., 2016) and another finding that only violent pornography use is predictive of sexual violence (Ferguson & Hartley, 2022). Although population studies typically find that the increased availability of pornography is related to lower rates of sexual aggression at the population level (Ferguson & Hartley, 2022), pornography use predicts increased sexual aggression in men with a history of aggression (Malamuth et al., 2000; Vega & Malamuth, 2007), suggesting that pornography use may only be associated with sexual aggression for men at risk of violence.

Other researchers have found that nonsexual physical aggression has been associated with pornography use. In a meta-analysis of 30 experimental studies (Allen et al., 1995), both men and women were found to have heightened rates of physical aggression after viewing pornography. Similarly, pornography use within romantic relationships has been related to negative outcomes for some couples. For instance, pornography users reported lower relationship quality and dedication, more negative communication, and lower relational adjustment compared to individuals who never viewed pornography (Maddox et al., 2011; Manning, 2006). Women who used higher rates of pornography held more negative self-perceptions than women who used less pornography (Daneback et al., 2009). For men, high pornography use was related to sexual aggression, lower relationship commitment, less fidelity, and problems with sexual arousal (Daneback et al., 2009; Lambert et al., 2012). However, in a longitudinal study of 1,234 people in heterosexual romantic relationships, watching pornography alone was associated with better relationship quality (e.g., relationship adjustment, emotional intimacy) for women and poorer relationship quality for men, and watching it together was associated with increases in sexual intimacy over time (Huntington et al., 2020). Interestingly, a dyadic study of 265 different-sex couples using an actor–partner interdependence model found that women’s higher individual pornography use frequency was related to higher rates of sexual activity with their partner (Bőthe, Vaillancourt-Morel, & Bergeron, 2021). Also, if women viewed pornography together with their partners, they tended to have higher levels of sexual function and lower sexual distress. In another study (*N* = 14,581 adults from Hungary), Bőthe,Tóth-Király (2021a) found that problematic pornography use moderately predicted sexual function problems in men and women; however, frequent pornography use had weak, negative links to sexual function problems for both men and women.

Minimal research has examined if pornography use is related to IPV. Of the few studies that do exist, most have been based on samples of either female residents in domestic violence shelters or men in batterer intervention programs (Brem et al., 2018; Shope, 2004; Simmons et al., 2008). Thus, results from these studies may not generalize to nonclinical populations. Rothman and Adhia (2016) conducted a study with primarily Black and Hispanic youth and found that IPV victimization was related to more frequent pornography use, viewing pornography with other people, and being asked to perform sex acts that partners saw in pornography. In the study with heterosexual couples by Huntington and colleagues (2020) mentioned previously, pornography use was associated with higher psychological aggression between partners. However, in a recent study using a sample of 892 university students, Hatch et al. (2020) found that pornography use did not prospectively predict perpetration of physical IPV 3 months later. This study used a cross-lagged panel design but did not include couple dyads. It did not account for potential dyadic effects at play in the relationship between pornography use and IPV. Thus, additional research is needed on the association between pornography use and IPV in community samples of couples.

### The Current Study

In the current study, we explored whether pornography use contributes to IPV while accounting for romantic partners’ mutual influence on each other and their baseline levels of pornography use. Using a longitudinal dyadic design, we examined the relation between frequency of pornography use (FPU) and IPV across a 4-month span in different-sex (i.e., male–female) couple dyads. This allowed for predicting changes in IPV over time from baseline FPU. Based on past research and theory, we hypothesized that high FPU among men at baseline would predict higher levels of IPV perpetration for men and higher levels of IPV victimization among their female partners 4 months later. Given the submissive and objectified depiction of women in mainstream pornography (Klaassen & Peter, 2015), it is plausible that women who view pornography might be cognitively primed to expect violence and to view themselves as sexual objects to be dominated based on the cognitive neoassociationistic model (Berkowitz, 1993), which posits that consuming violent or sexual media primes aggression-related cognitive constructs that subsequently become more accessible when interpreting environmental stimuli. Furthermore, women who view pornography tend to have a greater total number of sexual partners (Poulsen et al., 2013), which is associated with increased risk of IPV victimization (Gover, 2004). In light of this, we hypothesized that high FPU among women at baseline would predict higher levels of IPV victimization among women and higher levels of IPV perpetration for their male partners 4 months later. We only recruited different-sex couples in the study because relevant theory (i.e., the confluence model), most existing research, and our hypotheses concerned different-sex intimate relationships and our statistical analysis required that the couple dyads be distinguishable (e.g., by sex). Consistent with the majority of past literature, we defined pornography as explicit material that depicts or describes sexual subjects or activity that is mainly intended as a means of sexual arousal (Sneed, 2006), which could include pornographic magazine and films, erotic novels, sex tapes, and nude photos.

## Methods

### Participants

The final sample consisted of 132 different-sex couples (*N* = 264) in committed romantic relationships of at least 2 months. Participants ranged from 17 to 54 (*M* = 21.71, *SD* = 5.26) years old. In the final sample, 81.1% (*n* = 214) described themselves as White, 7.6% (*n* = 20) as Arabic/Middle Eastern, 4.2% (*n* = 11) as East Asian, 2.3% (*n* = 6) as Black/African Canadian, 2.3% (*n* = 6) as Mixed/Multiracial, 0.8% (*n* = 2) as Hispanic/Latino, 0.4% (*n* = 1) as Indigenous/First Nations/Metis, and 0.4% (*n* = 1) did not report their ethnicity. Most participants identified as Catholic (34.1%) or Atheist (31.8%) and lived with their parents (64.8%), romantic partner (18.2%), or roommates (10.6%). On average, participants started dating at the age of 17.97 years old (*SD* = 3.06, range = 11–33) with their average romantic relationship lasting 17.48 months (*SD* = 21.14, range = 1–184). Twenty-two percent (*n* = 58) disclosed experiencing IPV in the past. Participants’ current romantic relationships varied in length from 2 months to 25 years (*M* = 28.87 months, *SD* = 39.49) and were most commonly described as committed/exclusive dating (90.2%), followed by married (5.3%), engaged (3.8%), and casual dating (0.8%). Ninety percent of participants were sexually active in their current romantic relationship. On average, 17.4% of couples indicated that their relationship may end within the next 4 months.

### Measures

#### Demographic Information

Participants completed a self-report demographics questionnaire at both Time 1 and 2 of the study, which asked participants about their age, sex, gender, sexual orientation, ethnicity, level of education, religious affiliation, living situation, and socioeconomic status. It also included questions about participants’ intimate relationship history, current romantic relationship, and history of IPV. The demographic questionnaire for Time 2 (T2) of the study was slightly shorter than the one at Time 1 (T1) as it did not repeat questions asked at T1 pertaining to relatively stable demographic characteristics (i.e., ethnicity, religion, and education).

### Frequency of Pornography Use

There are no known existing validated measures of pornography use frequency for both men and women. We thus included two measures of pornography use with FPU subscales in our study, namely the Pornography Consumption Questionnaire (PCQ; Hald, 2006) and the Pornography Use Scale (PUS; Szymanski & Stewart-Richardson, 2014) to create a composite measure of FPU. The PCQ is an 86-item self-report measure of pornography use (Hald, 2006; Hald & Malamuth, 2008) that measures age of first exposure, FPU, pornography content preferences, financial impact of pornography consumption, sexual behavior, and realism of pornography. The PCQ’s FPU subscale has been found to load onto a single, continuous pornography use factor for both men and women (Hald, 2006), but it has not yet been formally evaluated psychometrically (Hald, et al., 2013; Hald & Mulya, 2013). We excluded the item “On average, how much time a week have you used to watch some kind of pornography during the last 4 months” from analyses because it substantially decreased the internal reliability of the scale at both T1 and T2 (α increased from .02 to .79 and from .03 to .81, respectively, after the item was removed). There was evidence that the item wording was confusing; responses widely varied and were often inconsistent with their reported pornography use frequency on other items. The PUS is a 14-item self-report measure of FPU and problematic pornography use (Szymanski & Stewart-Richardson, 2014). It contains a 7-item Frequency of Pornography Use (FPU) subscale that has been found to have good reliability among men (α = 0.88), but has not been evaluated with women. In this study, the PUS FPU scale had good internal consistency for both men (α_T1_ = .87, α_T2_ = .86) and women (α_T1_ = .87, α_T2_ = .88). Given that the PCQ FPU and PUS FPU subscales were strongly correlated (Spearman’s rank-order *ρ*_T1_ = .89; *ρ*_T2_ =.91) and collectively assessed a wider range of behaviors, we summed the standardized scores of the PCQ and PUS items measuring FPU to create a composite measure. The FPU composite items assess exposure patterns of pornography, frequency of pornography use, hours per week of pornography use, and amount of time spent using pornography per sitting (see Hald, 2006 and Szymanski & Stewart-Richardson, 2014 for a complete description of each item). The composite had excellent internal consistency (α_T1_ = .92, α_T2_ = .93) and good psychometrics (Jongsma, 2019).

### Intimate Partner Violence

We used the 78-item Revised Conflict Tactics Scales (CTS2; Straus et al., 1996) to measure the frequency with which men and women committed (39 items) and experienced (39 items) each act of physical, psychological, and sexual IPV within the preceding 4 months with their current partner using seven response options: *never* (0), *1 time* (1), *2 times* (2), *3–5 times* (4), *6–10 times* (8), *11–20 times* (15), or *more than 20 times* (25). Cronbach’s alphas for CTS2 scales have ranged from .79 to .95 (Straus et al., 1996). We calculated separate total sum scores for IPV perpetration and victimization for both men and women to limit the number of analyses and to ensure that any changes in IPV from T1 to T2 would be accounted for (e.g., decrease in physical IPV in conjunction with increase in psychological IPV). For men, internal reliabilities of the IPV perpetration and victimization composites were .66 and .63, respectively, at T1 and .81 and .79, respectively, at T2. Women’s internal reliabilities of IPV perpetration and victimization were .58 and .65, respectively, at T1 and .76 and .80, respectively, at T2.

### Social Desirability

As individuals often underreport their aggressive behaviors (Dutton & Hemphill, 1992; Saunders, 1991), we included the Marlowe-Crowne Social Desirability Short-Form C (MCSDS Form C; Reynolds, 1982) as a potential covariate. It consists of 13 true (1) and false (0) statements (e.g., “I am always courteous, even to people who are disagreeable”), with eight reverse-coded items. We summed all scores to create a total MCSDS score, such that higher scores represent more socially desirable responding. The MCSDS Form C has been found to have acceptable internal reliability (Kuder–Richardson 20 [KR-20] = .76; Reynolds, 1982), but it had questionable internal reliability in the current study at both T1 and T2 (KR-20 = .66 and .64, respectively).

### Validity Questions

We included one validity check question embedded in each questionnaire (*n* = 11) in both surveys to determine if participants were adequately attending to the task (e.g., “By reading this question, you will know that the answer is response four”). At the end of both surveys, we also included the following *yes*/*no* validity questions: (a) “Did you answer all of the questions honestly?”; (b) “Did you and your partner fill the surveys out separately?”; (c) “Do you have reason to believe that your survey results should not be included in this study?”.

### Procedures

Following clearance from the authors’ institutional research ethics board, students in intimate relationships attending a Canadian university were invited to participate in an online longitudinal study via the Psychology Participant Pool (a group of research participants who receive extra credit in eligible courses in exchange for participating in research) with their romantic partners. Interested individuals provided the name and contact information of their romantic partner. Both partners were then contacted separately, sent the study’s T1 survey URL as well as couple and individual identification numbers, and instructed to complete the survey separate from their partners. Participants completed the demographics questionnaire first, then the remaining questionnaires in a randomized order, and finally the three end-of-survey validity questions. Upon completion, participants were told that they would be re-contacted in 4 months for the T2 assessment and were presented with a list of community resources and instructions for clearing their Internet browser history (for safety purposes). Participants received a bonus point toward an eligible academic course or a $15 Amazon e-gift card for completing the survey. We re-contacted members of dyads for whom both partners completed T1 surveys 4 months (±1 week) after they had completed the T1 surveys to invite them to participate in T2 assessments. T2 procedures were identical to those at T1 except (a) participants were asked if they were still in a romantic relationship with their partner from T1 at the beginning of the T2 survey and (b) a shortened demographics questionnaire was administered.

A total of 679 participants completed the T1 survey. Cases were excluded if they did not have data from the corresponding partner (*n* = 113), leaving 283 couple dyads (*N* = 566) for whom both partners completed the T1 survey. Only these couples were invited to participate in T2 4 months later. Eleven of these dyads had ended their romantic relationships and were not eligible to participate at T2. A total of 342 participants also completed the T2 survey, but 46 cases had to be removed because there were no data from the corresponding partner, which left 148 dyads for whom both partners completed both T1 and T2 surveys. There was a 47.70% rate of attrition, and comparisons between those who dropped out after T1 did not reveal any significant differences from those who remained (Jongsma, 2019). Couple dyads were retained if each partner completed the survey, correctly answered over 70% of the embedded validity questions, and did not indicate that their responses were invalid via the standalone validity questions. Of the 283 dyads who completed the T1 survey, there were 34 individuals who did not meet these validity criteria (including five couples for whom both partners had invalid T1 data), which left 254 couples who completed the T1 survey and were deemed to have valid responses. For the 148 couples who also completed the T2 survey, 12 participants did not meet the validity criteria, which left 136 couples who completed the T2 survey and had valid responses. Overall, there were 132 couples (*N* = 264) who completed both T1 and T2 surveys and met the validity criteria at both T1 and T2, and these couple dyads were included in the longitudinal analyses.

### Statistical Analyses

**Figure 1.**
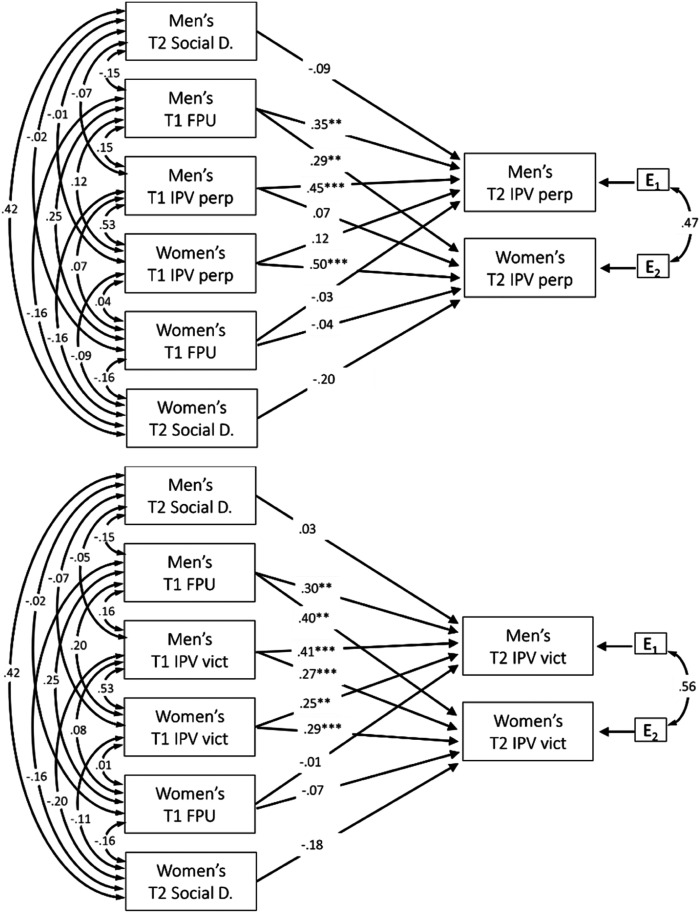
Unconstrained autoregressive models predicting Time 2 intimate partner violence (IPV) perpetration and victimization from Time 1 IPV perpetration and victimization and frequency of pornography use controlling for socially desirable responding. Note. Illustrates Model 4. Time 1 IPV perpetration (top panel) and victimization (bottom panel), Time 1 FPC, and Time 2 social desirability predicting Time 2 IPV perpetration (top panel) and victimization (bottom panel). Standardized coefficients (β) are presented for actor and partner effects. 132 couple dyads (*N* = 264). T1 = Time 1; T2 = Time 2; Social D. = social desirability; FPU = frequency of pornography use; IPV = intimate partner violence; perp = perpetration; vict =victimization; E_1_ = men’s error variance; E_2_ = women’s error variance*.* *p< .05. **p< .01. ***p< .001.

We analyzed the longitudinal dyadic data using the actor–partner interdependence model (APIM; Kenny et al., 2006) and separate path analysis models for perpetration and victimization (see Figure 1) in Mplus (version 8.0). Poisson regression was estimated via maximum likelihood robust estimation (MLR). In a longitudinal APIM, autoregressive effects describe the stability of the variables and the residual change in scores can be predicted by controlling for variable stability (Hartl et al., 2015; Popp et al., 2008). We were unable to calculate fit statistics as our dependent variables (i.e., IPV perpetration and victimization) were non-normal, count data (Muthén, 2009). We instead determined model fit using loglikelihood ratio chi-square difference testing (Asparouhov & Muthen, 2006). This involved comparing nested models containing the same variables with different degrees of free parameters to determine if the fit of the hypothesized model (Figure 1) was significantly better than a baseline, “null” model in which all estimated path coefficients were constrained to zero. For models that were not nested or contained different variables, we compared Akaike Information Criterions (AICs) and Bayes Information Criterions (BICs) between models to determine fit, with low AIC and BIC denoting better fit (Dziak et al., 2012; Kline, 2016).

## Results

### Preliminary Analyses

#### Missing Data, Tests of Nonindependence, and Distinguishability

There were no missing data for the sample of 132 couple dyads. However, there was nonindependence between partners’ scores. Spearman’s rank-order correlations between romantic partners’ reports of IPV perpetration and victimization at both T1 (*ρ* = .45 and *ρ* = .59, *p*s < .001, respectively) and T2 (*ρ* = .47 and *ρ* = .57, *p*s < .001, respectively) confirmed that respondents’ rates of IPV perpetration and victimization were related to those of their partners’, suggesting the need to analyze the data at the couple-level. In terms of distinguishability, given that members of the dyads differed by sex, distinguishability was established theoretically. To assess distinguishability empirically, we used Gonzalez and Griffin’s (1999) procedure for conducting an omnibus test of distinguishability with a saturated model using structural equation modeling in AMOS (Version 25). At T1, sex was a significant distinguishing factor in the model containing FPU and IPV perpetration, χ^2^(4, *N* = 132) = 21.9*, p*< .001, and the model with FPU and IPV victimization, χ^2^(4, *N* = 132) = 25.3, *p*< .001. Similarly, at T2, both models containing FPU and IPV perpetration, χ^2^(4, *N* = 132) = 13.7, *p* = 0.008, and FPU and IPV victimization, χ^2^(4, *N* = 132) = 12.6, *p* = 0.013, were distinguishable by sex. We therefore treated the dyads as distinguishable by sex.

### Descriptive Statistics and Bivariate Correlations

Table 1 presents descriptive statistics for key variables for men and women. Mean differences were tested with Wilcoxon signed rank sum tests for non-normal data. Men’s overall FPU composite was higher than that of women at both T1 and T2. About 50% of women and over 80% of men used pornography in the prior 4 months. For those who used pornography in the preceding 4 months, men reported using pornography 1–2 times per week at both T1 and T2, whereas women viewed pornography less than once a month at T1 and T2. Of those who disclosed using pornography, most men and women reported viewing pornography for less than 15 minutes per sitting. Men spent about 1.5 hours using pornography per week, which was significantly more than women’s roughly 0.5 hour per week. Men’s FPU did not significantly change over time, whereas women’s FPU decreased from T1 to T2. Both men’s and women’s socially desirable responding reduced from T1 to T2.

**Table 1.** Descriptive Statistics for Key Variables at Time 1 and Time 2.

	Time 1			Time 2		*z*(*df* = 132)	
	Mean *(SD)*	%		Mean *(SD)*	%		
Men							
Total IPV perp	9.58 (18.11)	62.1		7.82 (21.68)	54.5		−2.12*
Total IPV vict	9.51 (16.38)	62.9		8.70 (22.25)	56.8		−1.14
FPU	4.58 (8.84)	81.1		4.03 (8.29)	83.3		−0.70
Social desirability	6.22 (2.79)			4.38 (2.79)			−6.17***
Women							
Total IPV perp	9.41 (16.02)	61.4		8.61 (20.31)	54.5		−1.31
Total IPV vict	10.63 (19.20)	53.0		9.45 (22.98)	53.0		−1.41
FPU	−3.34 (6.05)	48.5		−3.87 (6.20)	50.8		−2.08*
Social desirability	6.22 (2.79)			4.38 (2.79)			−6.17***

*Note.* Total IPV perp = total frequency of acts of intimate partner violence perpetration; Total IPV vict = total frequency of acts of intimate partner violence victimization; FPU = frequency of pornography use composite; % = percentage of participants who endorsed at least one item.

* *p*< .05.** *p*< .01.*** *p*< .001.

We conducted a series of within-male (below diagonal), within-female (above diagonal), and interpartner (i.e., between dyad members) Spearman’s rank correlations to examine whether there were significant relations among key variables included in the longitudinal analyses (Table 2). Interpartner correlations indicate that there was nonindependence of observations and moderate agreement between dyad members. However, other interpartner agreement indices suggested relatively poor levels of interpartner agreement for male- (T1*k* = .13, T2*k* = .17) and female-perpetrated (T1*k* = .11, T2*k* = .12) IPV. Given this, we used individual, self-reported IPV perpetration and victimization data instead of aggregate scores for each couple. When assessing social desirability as a covariate, we found both T1 and T2 social desirability to be significantly related to FPU and IPV perpetration and victimization. We controlled for T2 social desirability in the longitudinal APIMs predicting T2 IPV given the lack of research on the predictive validity of social desirability on future aggression ratings.

**Table 2.** Within-Male, Within-Female, and Interpartner Correlations among Key Variables at Time 1 and Time 2.

Variable	1	2	3	4	5	6	7	8
1. Time 1 FPU	**.06**	.68***	.12	.11	.08	.07	−.22*	−.14
2. Time 2 FPU	.77***	.13*	.08	.15	.09	.14	−.29**	−.23**
3. Time 1 total IPV perp	.31***	.15	.45***	.71***	.87***	.70***	−.32***	−.16
4. Time 2 total IPV perp	.36***	.28**	.62***	.47***	.62***	.91***	−.29**	−.22*
5. Time 1 total IPV vict	.26**	.11	.91***	.60***	.59***	.67***	−.26**	−.14
6. Time 2 total IPV vict	.33***	.25**	.55***	.86***	.60***	.57***	−.19**	.17*
7. Time 1 social desirability	−.22*	−.21*	−.35***	−.39***	−.30**	−.32***	.21**	.51***
8. Time 2 social desirability	−.11	−.18*	−.15	−.24**	.12	.19*	.42***	.45***

*Note.* In the matrix, correlation for men appears below the diagonal and correlations for women appear above the diagonal. Bolded values along the diagonal are correlations between dyad members. FPU = frequency of pornography use; IPV = intimate partner violence; perp = perpetration; vict = victimization.

*p< .05.**p< .01.***p< .001.

### Main Analyses

For each type of IPV being predicted (i.e., T2 perpetration and T2 victimization), four models used the maximum likelihood robust (MLR) estimator correction, which is robust to non-normality (Muthén et al., 2017). Fit statistics for all eight models are displayed in Table 3. Models predicted T2 IPV perpetration/victimization from T1 IPV perpetration/victimization and T1 FPU and were tested with and without the T2 social desirability variable as a covariate. Model 1 is the null model (i.e., coefficients were set to zero) for the hypothesized model that did not include T2 social desirability. Model 2 is the hypothesized, unconstrained model (i.e., allowed male and female path coefficients to vary freely) that did not include T2 social desirability. Model 3 is the null model for the hypothesized model that included T2 social desirability, and Model 4 is the hypothesized, unconstrained model that included T2 social desirability. Because we could not compare models with versus without T2 social desirability using the loglikelihood ratio chi-square difference testing due to the difference in variables in the models, we examined AICs and BICs to determine the best fitting models.

**Table 3.** Model Fit Statistics for Intimate Partner Violence (IPV) Measurement Models.

Model	H Loglikelihood	Number Free Parameters	AIC	BIC	Adjusted BIC
IPV perpetration					
1	−892.69	5	1795.37	1809.78	1793.97
2	−747.09	13	1520.17	1557.65	1516.53
3	−892.69	5	1795.37	1809.78	1793.96
4	−733.98	15	1497.96	1541.20	1493.75
IPV victimization					
1	−894.34	5	1798.67	1813.09	1797.27
2	−815.71	13	1657.41	1694.89	1653.77
3	−894.34	5	1798.67	1813.09	1797.27
4	−800.18	15	1630.35	1673.60	1626.15

*Note.* AIC = Akaike Information Criterion; BIC = Bayes Information Criterion; IPV = intimate partner violence.

*p< .05.**p< .01.***p< .001.

### IPV Perpetration

Examination of AICs and BICs suggested that Model 4 (Figure 1), the unconstrained model that included T2 social desirability, was the best fitting model for T2 IPV perpetration. Loglikelihood chi-square difference testing indicated that Model 4 was a significantly better fit than its null model, Model 3, χ^2^(10, *N* = 132) = 317.41, *p*< .001. In line with our hypothesis concerning male IPV perpetration, frequent pornography use among men at T1 was associated with more acts of male-perpetrated IPV at T2 while controlling for men’s level of IPV perpetration at T1. Specifically, a standard deviation increase in FPU at T1 was associated with an increase in T2 male-perpetrated IPV of about one third of a standard deviation (β = .35, *p* = .003, 95% CI = .16–.55). As expected, men’s and women’s levels of IPV perpetration at T1 predicted their rate of IPV perpetration at T2 such that a standard deviation increase in T1 IPV was related to a .45-.50 standard deviation increase in men’s and women’s T2 IPV (men: β = .45, *p*< .001, 95% CI = .32–.59; women: β = .50, *p*< .001, 95% CI = .34–.66). When considering partner effects, contrary to our hypothesis that more frequent pornography use among women at T1 would be related to higher levels of T2 male-perpetrated IPV perpetration, women’s FPU at T1 was not significantly related to men’s IPV perpetration at T2 (β =−.03, *p* = .80, 95% CI =−.20-.15). However, if men frequently used pornography at T1, their female partners tended to perpetrate higher levels of IPV at T2 (β = .29, *p* = .007, 95% CI = .12–.47) at a rate of roughly a one third standard deviation increase in women’s T2 IPV for every standard deviation increase in men’s T1 FPU.

### IPV Victimization

Based on AICs and BICs, the best fitting model for T2 IPV victimization was Model 4 (Figure 1), which was unconstrained and controlled for T2 social desirability. Loglikelihood chi-square difference testing indicated that Model 4 was a significantly better fit than its null model, Model 3, χ^2^(10, *N* = 132) = 188.32, *p*< .001. As predicted, women tended to experience higher levels of IPV victimization at T2 if their male partners used pornography frequently at T1. Women’s IPV victimization at T2 increased .40 of a standard deviation for every unit increase in men’s pornography use at T1 (β =.40, *p* = .001, 95% CI = .20–.60). Contrary to our hypothesis that women with more frequent pornography use at T1 would have higher rates of IPV victimization at T2, women’s FPU at T1 did not predict their IPV victimization at T2 (β =−.07, *p* = .488, 95% CI =−.24-.10). Frequent pornography use among men at T1 was related to higher rates of IPV victimization for men at T2 when controlling for T1 IPV victimization and T2 social desirability at a rate of nearly one third of a standard deviation in men’s T2 IPV victimization for every unit increase in men’s T1 FPU (β = .30, *p* = .008, 95% CI = .12–.49). As anticipated, T1 IPV victimization predicted T2 IPV victimization for both men (β = .41, *p*< .001, 95% CI = .30–.53) and women (β = .29, *p*< .001, 95% CI = .17–.42) across the 4-month interval. Although not found for IPV perpetration, higher reports of men’s (β = .27, *p*< .001, 95% CI = .17–.38) and women’s (β = .25, *p* = .006, 95% CI = .10–.39) T1 IPV victimization were related to higher reports of their partners’ T2 IPV victimization.

## Discussion

This study prospectively examined how FPU in different-sex couples was related to changes in IPV perpetration and victimization over a 4-month period. Overall, if men frequently used pornography at baseline, over time, both male and female partners developed higher rates of IPV perpetration and victimization when initial levels of IPV and T2 socially desirable responding were controlled. This was consistent with our predictions of how men’s pornography use would impact IPV based on the cognitive neoassociationistic perspective (Berkowitz, 1993). However, contrary to expectations, women’s FPU did not predict changes in couples’ IPV over time. Not only do results provide evidence that frequent pornography use in men is a risk factor for IPV, the longitudinal design allowed us to demonstrate that high FPU among men resulted in increases in IPV over time. This provides some information about the direction of the relation and indicates that pornography use affects how IPV develops over time. In addition, the novel finding that couples developed higher rates of IPV over time if male partners frequently used pornography at baseline has meaningful implications for young people in different-sex romantic relationships given the widespread use of pornography particularly among men and the well-documented detrimental effects of IPV. However, a recent study has found that not all high frequency pornography use appears to be problematic (Bőthe et al., 2020), so perhaps FPU may only be associated with higher rates of IPV perpetration for only a subset of men. This is consistent with Malamuth’s (1995) confluence model which theorizes that pornography use contributes to sexual aggression for men who are predisposed to sexual violence.

When considering why frequent pornography use among men but not women predicted increases in IPV, this may be because women used pornography much less frequently than men, rather than there being a qualitative difference between men and women that would make pornography use more risky for men than women. However, we cannot rule out the possibility that women might be less vulnerable to the impact that pornography has on IPV, which could be similar to Huntington’s et al. (2020) findings that watching pornography alone was related to better relationship quality for women and poorer relationship quality for men.

In terms of how variables of interest changed over time, men’s and women’s FPU and IPV perpetration and victimization tended to slightly decline from T1 to T2. Though most of these differences were not statistically significant, men’s self-reported IPV perpetration significantly decreased over time. Despite this, women’s IPV victimization did not significantly reduce in kind. However, significant reductions were found in women’s FPU over time. Item-level analysis of the reductions in women’s FPU from T1 to T2 indicated that a similar proportion of women were consuming pornography at T2, but those who used pornography reported viewing it on fewer occasions and for shorter periods of time than at baseline. These decreases in FPU and IPV from T1 to T2 might be a consequence of couples participating in T1 of the study as they were asked extensive questions about their pornography use and IPV at T1 (i.e., testing effects). As a result, participants may have reflected on their pornography use and IPV and made efforts to decrease their pornography use and IPV.

### Limitations and Future Directions

This study has notable limitations. First, the findings are limited by the study’s dependence on self-report measures and the biases inherent with self-report. For instance, participants who reported fewer acts of IPV on the CTS2 tended to present themselves in a more socially desirable manner. We thus controlled for social desirability in analyses. As is typical (Marshall et al., 2020), couples generally had low levels of interpartner agreement in reports of IPV, which highlights the difficulties with obtaining accurate responses when assessing aggression via self-reports retrospectively. To address these limitations, researchers are recommended to develop multimodal methods of assessment. Future studies could draw on technology to prospectively track participants’ online pornography use with the use of a mobile app or computer program, which would likely improve the accuracy of measuring FPU compared to self-report.

Second, given the limited availability of reliable measures of FPU, the current study used a composite of two pornography questionnaires to measure FPU to maximize the reliability of the measure. This highlights the need for researchers to develop psychometrically sound measures of pornography use for both men and women.

Third, the T1 IPV and T1 and T2 social desirability measures used in this study had questionable reliability, which could have reduced the strength of the models and the ability to identify significant effects (Kline, 2016). The limited reliability of the CTS2 has been shown to be common, especially among nonclinical samples (Lorber & Slep, 2018; Wilson et al., 2018). The CTS2 is also limited in that it lacks information about the context and motivations of the IPV, which is relevant information for the interpretation of study findings. For instance, it was unclear if male partners who frequently used pornography initiated more violence toward their female partners or if female partners responded to their male partners’ heavy pornography use with increased IPV. In addition, we combined physical, sexual, and psychological IPV into composites for perpetration and victimization, but FPU may not be related to all three of these types of IPV or in the same way. Further research could examine these separately to better understand the relation between FPU and IPV subtypes.

Fourth, this study focused on the FPU, but did not discriminate between different types of pornography. There are many different genres of pornography that vary widely in their content, target audience, degree of violence, and depiction of men and women. It would not be surprising if the type of pornography being viewed influenced the relation between pornography use and IPV, and this will be an interesting area for future studies to explore. We speculate that the finding that high FPU among men (but not women) resulted in increases in IPV over time is related to differences in the types of pornography used by men versus women. Although categorically grouping pornography genres may be cumbersome and difficult to analyze, we suggest that researchers considering measuring the degree of violence of the pornography consumed as this metric may mediate the associations between men’s FPU and IPV. Another useful concept that should be incorporated into future studies is problematic pornography use, which recent research has found to be more closely related to negative outcomes than FPU in both men and women(Bőthe et al., 2021a, 2022).

This study did not account for whether partners viewed pornography alone or together. This is a key limitation as viewing pornography alone versus with a romantic partner has been found to be related to different outcomes (e.g., Bőthe et al., 2021a, 2022; Huntington et al., 2020). Also, we did not differentiate whether participants were being forced to view pornography, which would actually be a form of IPV and separate from consensual pornography use.

Another limitation of this study was the high attrition rate (47.7%), which was due, in part, to 11 couples breaking up, both partners being required to complete T1 to participate in T2, and attrition for other unknown reasons. Although it is possible that there were differences between those who completed both T1 and T2 of the study and those who did not, resulting in a self-selection bias that may have affected the findings of the study, those who completed both T1 and T2 and those who did not did not differ on any key variables measured at T1. Future studies may consider using shorter time intervals or ecological momentary assessments to reduce attrition.

Another important factor is that at least one partner in each couple was a university student enrolled in a psychology course. Moreover, generalizability of findings may be limited to White Canadian young adults from middle class backgrounds with a minimum of a high school education. Further, most participants were university students who lived with their parents, and most couples were dating and had been with their partner for about a year and a half. Therefore, it is unclear whether study findings can be generalized to same-sex couples or to different-sex couples from non-university samples or more varied cultural, socioeconomic, and educational backgrounds. Further research should focus on replication and extension to more diverse samples.

Due to the complexity of the longitudinal APIM models and the relatively small sample size, the only covariate included in this study was social desirability. We did not examine how pornography use influences IPV as there are likely a number of important latent factors (e.g., emotional regulation, experiential avoidance, interpersonal skills, sexual expectations, and relationship satisfaction) and interactive variables (e.g., sex drive, viewing pornography alone or with one’s partner) not accounted for in this study that future studies should explore. Researchers should consider possible gender differences in the way in which FPU affects IPV, which could help to better understand the nature of the relation between FPU and IPV for men versus women.

## Conclusions

This study addresses whether the burgeoning use of pornography in the internet age is harmful. The finding that couples developed higher rates of IPV over time if male partners frequently used pornography at T1 may have meaningful implications for the population at large given the widespread use of pornography, particularly among men, as well as the well-documented detrimental effects of IPV. However, the findings should be replicated with a more diverse sample (larger age range, larger average relationship duration, more co-habiting couples) before results are incorporated into recommendations to the public. If this were to happen, the evidence that frequent pornography use by men is a risk factor for IPV could bolster support for existing interventions for problematic pornography use, and this information could be included in the psychoeducational components of these interventions (Wéry & Billieux, 2017) as well as in IPV prevention and intervention initiatives. In addition, findings may suggest that couples therapists should consider assessing couples’ pornography use as well as its role in relationship conflict. Additional research is thus needed to inform best practices related to FPC and IPV.

This study demonstrates the importance of taking a couple-level approach to better understand risk and protective factors for IPV as interactions that take place between partners are key in determining why some couples resort to violence. It sheds light on the complex relations between FPU and IPV perpetration and victimization, but there is still much about these associations that are not well understood. Future research can build upon these findings to develop a more thorough understanding of the impact of men’s and women’s pornography use on IPV.
